# Spore Survival During Abrasive Saltation on Mars: A Comment on Bak *et al.*

**DOI:** 10.1089/ast.2021.0143

**Published:** 2022-09-05

**Authors:** Charles H. Minns, Emma M.C. Louden, Christopher F. Chyba

**Affiliations:** ^1^ Department of Physics and Astronomy, University College London, Gower Street, London, UK.; ^2^ Department of Astronomy, Yale University, New Haven, Connecticut, USA.; ^3^ Department of Astrophysical Sciences and School of Public and International Affairs, Princeton University, Princeton, New Jersey, USA.

**Keywords:** Aeolian saltation, Forward contamination, Martian habitability, Abrasion, Spores, Endospores

## Abstract

In original experiments, Bak *et al.* (Wind-Driven Saltation: An Overlooked Challenge for Life on Mars. *Astrobiology* 2019;19(4):497–505) suggest a new mechanism for the destruction of spores on Mars: abrasion by wind-driven saltation. Bak *et al.* found that the tumbling of spores on grain surfaces (simulating saltation) was, by far, most lethal at the outset of their experiments. They suggest that it may be sharp edges of the freshly crushed basalt particles used in their experiments that destroy the spores and that these edges abrade away over the course of each experiment. But prior Mars analogue experiments, observations of particles from terrestrial deserts, and imaging from Mars landers suggest that most martian dust has been rounded by billions of years of aeolian processes. If so, saltation on Mars is more likely well simulated by the later stages of the Bak *et al.* experiments, reducing implied lethality by orders of magnitude. Experiments could test this by beginning with particles that had been already abraded. Even assuming the highest lethality found in their experiments, saltation “hop” distances on Mars suggest that abrasion would not prevent ∼1% of released spores from remaining viable while traveling hundreds or even thousands of kilometers.

The current framework for the planetary protection of Mars rests upon the idea of “special regions” (Rummel *et al.,* 2014; National Academies of Sciences, Engineering, and Medicine, 2015). These are zones “within which terrestrial organisms are likely to propagate, or a region which is interpreted to have a high potential for the existence of extant martian life forms” (Committee on Space Research, 2003). Spacecraft that will have direct contact with special regions must undergo particularly stringent (Viking lander-level) bioload reduction. This instantiates the Outer Space Treaty's requirement that nations must avoid the “harmful contamination” of celestial bodies (Article IX) and that this (like all the Treaty's provisions) applies to the activities of both governmental agencies and nongovernmental entities within those nations (Article VI).

For this special-region approach to martian planetary protection to be credible, it must in fact be the case that it is difficult for viable microorganisms (possibly in the form of bacterial endospores) to survive wind-driven (via lofting or saltation) transport in the martian atmosphere across distances from any spacecraft landed in a non-special region to any special regions that lie downwind. Mars rovers are required to have no more than 300,000 spores in a position from which they could enter the martian environment (NASA, n.d.). A common expectation has been that aeolian transport from such a rover or other lander is likely to prove fatal for transported microorganisms, even spores, especially given the martian ultraviolet light environment (National Research Council, 2006).

In original and carefully controlled experiments, Bak *et al.* (2019) have suggested an important possible additional mechanism for the destruction of wind-blown spores on Mars. Following work by Jones *et al.* (2005) on the mechanical abrasion of spores, they identify abrasion by wind-driven saltation as a possible kill mechanism. To quantify this effect, Bak *et al.* used particles of Icelandic basalt freshly crushed into the size range 125 micron to 1 mm as an analogue for martian soil. Spores of *Bacillus subtilis* were coated onto the particles and then maintained under simulated martian atmospheric composition and pressure. One sample was placed in a tumbling system to simulate saltation, and another was used as a control group. Experiments showed that the simulated saltation significantly reduced the number of colony-forming units (CFUs) compared with a non-saltated control, and they concluded that this process “may efficiently protect the surface from forward contamination” (Bak *et al.* 2019).

Bak *et al.* found that “the number of viable spores decreased by about 50% within the first minute [of simulated saltation], while it took 5 days to inactivate the same percentage of the spores that already had survived 5 days of simulated saltation.” They present two hypotheses to explain this exponential increase in spore survival time. The first is that it is the sharp edges of the freshly crushed basalt that destroy the spores; as the experiment runs, the basalt particles are smoothed by abrasion and, therefore, become less and less lethal to the spores. Their second hypothesis is that spores may get trapped in cavities, so these spores will only be destroyed after the cavities crack open.

Consider the first of these hypotheses, that spore destruction is driven by the presence of sharp edges on the basalt particles. But such sharp edges seem unlikely to be present on typical grains on Mars, a planet where physical weathering has dominated the surface for the past 3 billion years (*e.g.,* Cornwall *et al.,* 2015). Indeed, laboratory simulations of abrasion of basalt grains by saltation on Mars found that initially angular particles abraded to rounded particles with “rather smooth” surfaces (Krinsley *et al.,* 1979). Similar conclusions come from the study of terrestrial grains in dry deserts (Cornwall *et al.,* 2015), images of dust from the Phoenix lander (“lithic fragments … rounded by eolian transport”; Goetz *et al.,* 2010), and from the Curiosity rover's Mars Hand Lens Imager (MAHLI) (where the majority of the resolvable grains less than about 150 microns in size were “sub-rounded to rounded,” though a minor component maintained angular shapes; Minitti *et al.,* 2013). This suggests that more realistic simulations would begin with grains that had already been smoothed by abrasive tumbling before the spores were introduced. The data presented by Bak *et al.* suggest that this could lengthen the survival time of spores in their experiments by several orders of magnitude. Experiments could be run with grains that had been tumbled for different lengths of time prior to the introduction of spores. We say this while being conscious of the fact that it is easy for an outside observer to propose additional experiments, while it is challenging, time-consuming, and possibly expensive to actually perform them.

Finally, we note that even the current Bak *et al.* data suggest that 100-micron saltating particles could travel extensive distances on Mars before being abrasively sterilized. If we follow Bak *et al.* and take one experimental rotation collision to correspond to one saltation hop, we can use models for saltation hop distance to estimate the transport distance that corresponds to a certain number of rotations (saltation hops). We initially envision these hops occurring under the influence of a prevailing wind, so that the appropriate simulation is not a random walk but rather closer to a linear addition of hop distances—an assumption that clearly gives a maximum possible transportation distance. Almeida *et al.* (2008) gave the saltation hop distance $L_{salt}$ on a planetary surface to be

$$L_{salt}\approx 1091.5\cdot {\left(\frac{v^{2}}{g}\right)}^{\frac{1}{3}}\cdot \frac{\left(u_{*}-u_{*t}\right)}{\sqrt{gd}}$$

where *v* is $\frac{\eta }{\rho }$, where η is the fluid viscosity and ρ is the fluid density, *g* is surface gravity, *d* is particle diameter, $u_{*}$ is the shear velocity, and $u_{*t}$ is the impact threshold wind speed. For Mars, η=1.3 × 10^−5^ kg m^−1^ s^−1^, ρ=0.02 kg m^−3^, *g* = 3.71 m s^−1^, $u_{*}$ is estimated to be in the range 1.12–1.78 m s^−1^, and $u_{*t}$ = 1.12 m s^−1^ (Almeida *et al.* 2008). We choose the average of this range, $u_{*}$ = 1.45 m s^−1^. Then $L_{salt}\approx$ 29 m for 1 mm diameter particles and 81 m for 0.125 mm particles.

In Table 1, we display experimental tumbling time and the corresponding number of rotations (saltation hops) from the Bak *et al.* experiments, as well as the percentages of viable spores remaining after this number of rotations (from our own digitization of the data in Fig. 2 of Bak *et al.*). For 1 and 0.125 mm particles, given the corresponding values of $L_{salt}$, we then show the maximum total distance that could be travelled by the surviving spores. This estimate is a maximum distance because it assumes that the saltation hops simply add linearly. That prevailing winds may move saltating grains in a roughly uniform direction seems plausible in light of observations of sand dune ripple migration (Silvestro *et al.,* 2010). However, as a minimum-migration end-member estimate, we also include distances travelled by saltating grains on the assumption that there is no prevailing wind direction, so that each saltation hop is in a random direction and saltating grains move by random walk with step size $L_{salt}.$

**Table 1.** Travel Distance and Survival for Saltating Spores on Mars

Tumbling time	Number of hops	Minimum (random walk) travel distance for 1 mm particle (km)	Maximum (linear) travel distance for 1 mm particle (km)	Minimum (random walk) travel distance for 0.125 mm particle (km)	Maximum (linear) travel distance for 0.125 mm particle (km)	Percentage of original spores remaining
1 minute	60	0.2	2	0.6	5	50%
1 hour	3,600	2	100	5	300	3%
1 day	86,400	8	2,000	24	7,000	0.5%

Results take into account only abrasion as a kill mechanism. Survival may be much higher than indicated here if martian dust, as seems likely, is much more rounded than the freshly crushed particles used in the Bak *et al.* experiments (see discussion in text).

If the primary kill mechanism for the spores in the Bak *et al.* experiments is abrasion by sharp edges on newly crushed basalt, Table 1 may greatly underestimate the percentage of surviving spores that corresponds to the travel distances shown, because actual martian saltating particles are likely to be much more rounded than those used in the experiments. The survival fraction shown is therefore a worst case for % survival. Of course, survival in Table 1 is calculated assuming that abrasion is the only kill mechanism operating during saltation on Mars; survival could instead be limited by other factors, such as damage by ultraviolet light. These factors require their own investigation.

## Conclusion

Bak *et al.* (2019) presented results from original and well-controlled experiments that suggest that abrasive saltation may be an important kill mechanism for wind-blown spores on Mars. However, they also note that the data suggest the possibility that the freshly crushed basalt used in their experiment as a Mars analogue becomes less lethal as repeated tumbling abrades away its original sharp edges. Other experiments with Mars analogues, observations of particles from dry deserts on Earth, and imaging from Mars landers suggest that most martian dust will be rounded and without sharp edges, due to billions of years of martian aeolian processes. If so, spore survival under saltation is more likely to be well simulated by the later stages of the Bak *et al.* experiments, in which lethality is reduced by several orders of magnitude. Experiments that begin with particles that had been abraded by tumbling prior to the introduction of spores could test this. It seems likely that abrasive saltation, by itself, will not prevent spores from surviving saltation over at least tens, more likely hundreds, and plausibly thousands of kilometers on Mars.
